# Apicoplast Lipoic Acid Protein Ligase B Is Not Essential for Plasmodium falciparum

**DOI:** 10.1371/journal.ppat.0030189

**Published:** 2007-12-07

**Authors:** Svenja Günther, Lynsey Wallace, Eva-Maria Patzewitz, Paul J McMillan, Janet Storm, Carsten Wrenger, Ryan Bissett, Terry K Smith, Sylke Müller

**Affiliations:** 1 Division of Infection and Immunity, Faculty of Biomedical and Life Sciences, University of Glasgow, Glasgow, United Kingdom; 2 Wellcome Centre for Parasitology, Glasgow, United Kingdom; 3 Bernhard Nocht Institute for Tropical Medicine, Hamburg, Germany; 4 Division of Biological Chemistry and Drug Discovery, College of Life Sciences, University of Dundee, Dundee, United Kingdom; Washington University School of Medicine, United States of America

## Abstract

Lipoic acid (LA) is an essential cofactor of α-keto acid dehydrogenase complexes (KADHs) and the glycine cleavage system. In *Plasmodium*, LA is attached to the KADHs by organelle-specific lipoylation pathways. Biosynthesis of LA exclusively occurs in the apicoplast, comprising octanoyl-[acyl carrier protein]: protein N-octanoyltransferase (LipB) and LA synthase. Salvage of LA is mitochondrial and scavenged LA is ligated to the KADHs by LA protein ligase 1 (LplA1). Both pathways are entirely independent, suggesting that both are likely to be essential for parasite survival. However, disruption of the *LipB* gene did not negatively affect parasite growth despite a drastic loss of LA (>90%). Surprisingly, the sole, apicoplast-located pyruvate dehydrogenase still showed lipoylation, suggesting that an alternative lipoylation pathway exists in this organelle. We provide evidence that this residual lipoylation is attributable to the dual targeted, functional lipoate protein ligase 2 (LplA2). Localisation studies show that LplA2 is present in both mitochondrion and apicoplast suggesting redundancy between the lipoic acid protein ligases in the erythrocytic stages of P. falciparum.

## Author Summary

Plasmodium falciparum is the causative agent of severe malaria. The parasites possess two organelles that are integral to their metabolism—the mitochondrion and the apicoplast, a remnant plastid. Both organelles contain enzymes that depend on the attachment of the cofactor lipoic acid for their catalytic activity. These are the α-keto acid dehydrogenase complexes and the glycine cleavage system (GCS). The pyruvate dehydrogenase (PDH) is solely found in the apicoplast of the parasites whereas α-keto glutarate and branched chain α-keto acid dehydrogenase as well as the GCS are mitochondrial. Both organelles possess specific and independent mechanisms that guarantee the posttranslational lipoylation of these enzyme complexes. In this study we show that the apicoplast located lipoic acid protein ligase, octanoyl-[acyl carrier protein]: protein N-octanoyltransferase (LipB), is not essential for parasite survival by disrupting the *LipB* gene locus. Despite a drastic loss of total lipoic acid, the parasites progress through their intraerythrocytic development unperturbed although the apicoplast-located PDH shows a reduced level of lipoylation. This phenotype is attributable to the presence of the recently described lipoic acid protein ligase 2, LplA2, which we show to be dually targeted to mitochondrion and apicoplast.

## Introduction

Lipoic acid (6,8-thioctic acid; LA) is an essential cofactor that is covalently attached to the transacylase subunit (E2-subunit) of α-keto acid dehydrogenase complexes (KADHs), namely pyruvate dehydrogenase (PDH), α-keto glutarate dehydrogenase (KGDH), and branched chain α-keto acid dehydrogenase (BCDH) as well as the H-protein of the glycine cleavage system (GCS) [1,2]. In eukaryotes, these multienzyme complexes are generally found in the mitochondrion. Only plants and plastid-containing organisms possess organelle-specific PDH with the plastid PDH providing substrates for fatty acid biosynthesis [3]. Therefore, mitochondrion and plastid require the enzymatic machineries for the posttranslational lipoylation of KADHs or H-protein [2–5].

LA is provided and ligated to the respective target proteins by two distinct pathways. The cofactor can be synthesised by almost all organisms using the LA biosynthesis pathway. This requires octanoyl-acyl carrier protein (ACP) as a substrate (a product of fatty acid biosynthesis) which is ligated to the apo-E2-subunits or the apo-H-protein by octanoyl-[acyl carrier protein]: protein N-octanoyltransferase (LipB) [6]. Subsequently, two sulphurs are introduced into position 6 and 8 of the protein-bound octanoic acid, a reaction that is catalysed by lipoic acid synthase (LipA) [7,8]. LA can also be acquired through the salvage pathway. In mammals free, salvaged LA is transferred to the E2-subunits of KADHs through two enzymatic steps but in bacteria, fungi, and apicomplexan parasites this reaction is catalysed by a single enzyme [6,9–12]. Scavenged LA in mammals is first activated through an ATP-dependent reaction catalysed by LA activating enzyme before the activated form of LA is then attached to the E2-subunits or the H-protein by LA transferase [9,10]. In contrast, bacterial-type LA protein ligases (LplA) catalyse the activation and transfer of LA in a single enzymatic step [6].

LA metabolism in the malaria parasite Plasmodium falciparum and the related apicomplexan parasite Toxoplasma gondii display an organelle-specific distribution of biosynthetic and salvage pathways [11–15]. LA biosynthesis is exclusively found in their plastid-like organelle, the apicoplast, whereas LA salvage is confined to their mitochondrion. It was shown that both organelles contain members of the KADHs, which require posttranslational lipoylation [16–18]. It is assumed that these multienzyme complexes play pivotal roles in the parasite's metabolism and it is thought that both LA biosynthesis and salvage are essential for parasite survival. This is further supported by the findings of Crawford and colleagues [13] showing that newly synthesised LA does not exit the apicoplast and by Allary and colleagues [14] who showed that radiolabelled LA is not utilised to lipoylate apicoplast PDH, but only leads to lipoylation of the mitochondrial E2-subunits. These studies therefore provide evidence that the organelles' lipoylation machineries act independently and inhibition of either one should have deleterious effects for the parasites. Indeed proof of this concept is supported by the lethal effect of the LA analogue 8-bromo-octanoic acid on intraerythrocytic stages of P. falciparum and also T. gondii [13,14]. In this study we have further tested this hypothesis by disrupting the *Plasmodium* apicoplast targeted *LipB* gene, which is part of the LA de novo biosynthesis pathway. Surprisingly, the gene disruption is not deleterious for the parasites suggesting that *Plasmodium* possesses alternative routes for LA ligation in this organelle. Our results provide evidence that a second *LA* protein ligase-like protein, LplA2 [13,14], can replace LipB function.

## Results

### Plasmodium falciparum Possesses Lipoylated KADH and H-Protein

To verify that the protein bands recognised by an antibody directed against protein-bound LA (anti-LA), which was used in this study to detect lipoylated proteins in P. falciparum (Figure 1, lane 4), Western blots of parasite extracts were also analysed with antisera raised against P. falciparum H-protein, BCDH-E2 and KGDH-E2 (Figure 1, lanes 1–3). The sizes of the proteins detected by the anti-LA antibody correlated well with those protein bands detected by the antibodies directed against the three mitochondrial proteins. These results corroborate the previously published suggestion that the protein bands detected by the anti-LA antibody in fact represent the mitochondrial KADHs. The 75 kDa band that was detected by anti-LA was previously shown to correspond to the PDH-E2-subunit [14].

**Figure 1:**
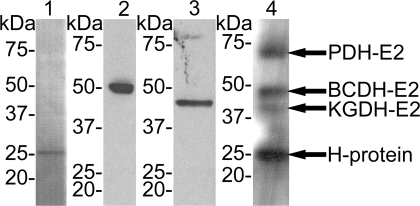
Identification of Lipoylated Proteins in P. falciparum P. falciparum protein extracts (15 μg) were separated on an SDS-PAGE (4%–12%, Invitrogen) and blotted onto nitrocellulose as described in the Materials and Methods section. The blots were analysed using four different antibodies: (lane 1) rabbit anti-H-protein at 1:2,000 recognising a 25 kDa band; (lane 2) rabbit anti-BCDH-E2 at 1:100 recognising a 50 kDa band; (lane 3) rat anti-KGDH-E2 at 1:5,000 recognising a 47 kDa band; (lane 4) rabbit anti-lipoic acid at 1:500 recognising four major protein bands corresponding to PDH-E2 (75 kDa, Allary et al. [14]), BCDH-E2 (50 kDa), KGDH-E2 (47 kDa) and H-protein (25 kDa).

### The *LipB* Gene Can Be Disrupted

The gene encoding LipB was targeted using two different constructs (see Materials and Methods) cloned into the P. falciparum transfection plasmid pHH1. This plasmid confers single cross over recombination resulting in a disruption of the endogenous gene locus [19]. Both constructs lacked the last 100 amino acids including a catalytically essential, highly conserved cysteine residue at position 369 (*Plasmodium* LipB numbering) which was shown to form a catalytic dyad with a conserved lysine residue in position 307 (*Plasmodium* LipB numbering) in the Mycobacterium tuberculosum LipB protein [20]. Therefore it was assumed that the disruption of the endogenous *LipB* gene locus should result in the generation of a C-terminally truncated LipB protein unlikely to display any enzymatic activity.

Independent transfections of both constructs were performed and the genotype of the transfected parasite lines was analysed by Southern blotting to verify the presence of the transfected plasmid. All analysed parasite genotypes revealed that the *LipB* gene locus had been targeted and the independent transfectants were cloned by limiting dilution. Two independent clones from separate transfections with each construct were used for further analyses (Figure 2). *LipB*KO1–1 and *LipB*KO1–2 describe the 3D7 derived mutants and *LipB*KO2–1 and *LipB*KO2–2 describe the D10 derived mutants. Upon NdeI digestion of genomic DNA of all four clones, the endogenous gene of 2.8 kb (Figure 2, lanes 1 and 2) is replaced by two bands of approximately 3.7 kb and 4.9 kb (Figure 2, lanes 3–6) diagnostic for the disruption of the *LipB* gene locus.

**Figure 2:**
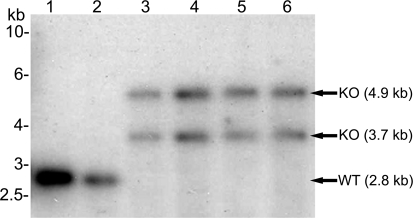
Southern Blot of *P. falciparum LipB* Mutants Genomic DNA was isolated from wild-type (P. falciparum 3D7 and D10) as well as four independent *LipB* mutant clones (KO1–1, KO1–2; KO2–1 and KO2–2). The DNA was digested with NdeI and separated on a 0.8% agarose gel before blotting to nylon and probing with the *LipB* coding region. The band shown in lanes 1 and 2 corresponds to the endogenous *LipB* gene locus (2.8 kb). The 2.8 kb band disappears and is replaced by two bands diagnostic for the disruption of the endogenous *LipB* locus (3.7 kb and 4.9 kb) in all four cloned parasite lines (lanes 3–6).

### Does the *LipB* Knockout Affect Parasite Growth?

Growth experiments performed according to Sanders et al. [21] showed that the *LipB* disruption with either construct, and regardless of parasite strain, resulted in a modestly increased growth rate of the *LipB* mutants compared to both parent lines (3D7 and D10) (Figure 3). Six days after the experiment was started with highly synchronised, ring-stage parasites, the parasitemia of the wild-type controls was determined to be between 1.8% and 2% whereas the *LipB* mutants consistently had a parasitemia between 4% and 5%. This increased growth rate could be explained in several ways; e.g., an accelerated cell cycle, the generation of a greater number of merozoites, or a more successful invasion rate by the mutant parasites.

**Figure 3:**
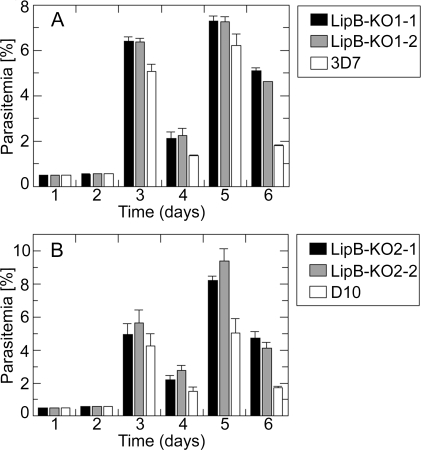
Growth of *LipB* Mutants Parasite growth was assessed over six days according to Sanders et al. [21]. (A) 3D7 wild-type and KO1–1 and KO1–2; (B) D10 wild-type and KO2–1 and KO2–2. The experiment was started with highly synchronised ring-staged cultures which were monitored daily and diluted 5-fold every 48 h. For each determination of percentage parasitemia, the number of infected erythrocytes per 1,000 erythrocytes was recorded. Means and standard errors of three separate experiments are shown.

The first point was addressed by following tightly synchronised parasites through the 48 h developmental cycle. Blood smears were taken every 8 h and the progression through the cell cycle of the different parasite lines was analysed microscopically (Figure 4). The experiment was started with tightly synchronised ring-stage parasites of all six parasite lines and this time point was set as 0 h. Eight hours later, between 50% and 80% of the *LipB* mutants had developed into trophozoites, whereas only 20% to 25% of either D10 or 3D7 *Plasmodium* had progressed to this developmental stage. Generally the 3D7 wild-type and the 3D7-based mutants showed a faster progression through their intraerythrocytic development than the D10 wild-type and mutant lines (Figure 4). Thirty two hours after the start of the experiment almost all of the *LipB* mutant parasites had re-infected fresh erythrocytes and had successfully progressed through their life cycle, whereas 3D7 needed another 8 h to reach this point and D10 needed even longer. In summary, the data clearly show that all four *LipB*KO mutants (the two selected 3D7-based KO1 clones and the two D10-based KO2 clones) progress through their intraerythrocytic cell cycle faster (about 4 to 8 h) than the two wild-type lines, which suggests that the disruption of the *LipB* gene locus in some way affects parasite cell cycle control. Determining parasite numbers at the beginning and the end of the parasites' life cycles revealed that the *LipB*KO schizonts generated, on average, the same number of successfully infective merozoites as wild-type parasites per life cycle.

**Figure 4:**
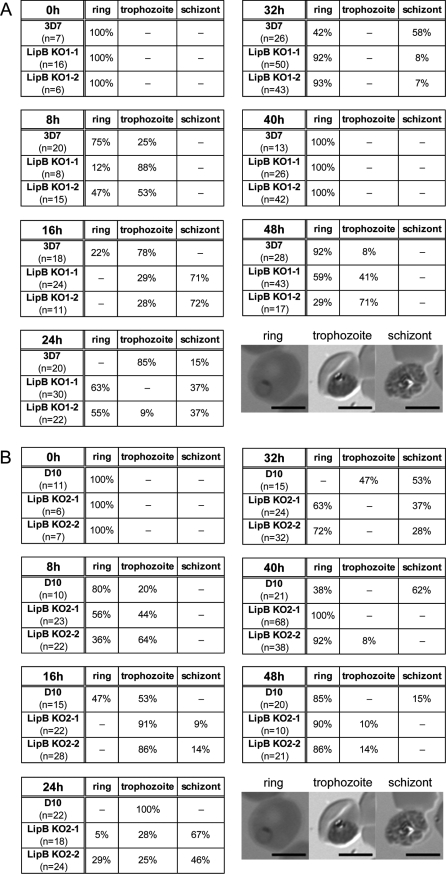
Progression of Wild-Type and *LipB*KO Mutants through Intraerythrocytic Cell Cycle Progression through the intraerythrocytic developmental cycle was assessed by analysing Giemsa stained thin-smears of highly synchronised cultures that were taken every 8 h for a period of 48 h. The experiments were started with ring-stage parasites (rings) and the development through one intraerythrocytic life cycle was followed by determining the occurrence of rings, trophozoites, and schizonts by light microscopy. The images represent typical appearance of these parasite stages. The numbers show the percentage of rings, trophozoites, and schizonts counted at the respective time point. The progression of the *LipB* mutants (3D7-based, panel A and D10-based, panel B) through the intraerythrocytic cell cycle is accelerated by about 4 to 8 h in comparison with wild-type 3D7 and D10. Scale bars: 5 μm.

### Effect on Lipoic Acid Content

The LA content of wild-type and the *LipB* null mutants was quantified by gas chromatography-mass spectrometry (GC-MS). This revealed that all of the *LipB* null mutants showed a drastic reduction in total LA content (Figure 5A; Table 1) compared to the wild-type parasite lines. Both P. falciparum 3D7 and D10 contain approximately 40 nmol/10^8^ cells of LA whereas the *LipB* mutants only contain between 0.6 nmol/10^8^ cells and 2.2 nmol/10^8^ cells; a reduction of more than 90% of total LA. In addition to the total LA in the cell, it was possible to distinguish between oxidised and reduced protein-bound LA; the ratio of which was only marginally affected by the *LipB* disruption, varying between 1.2 and 4.5, with the oxidised form of LA being predominant (Table 1).

**Figure 5:**
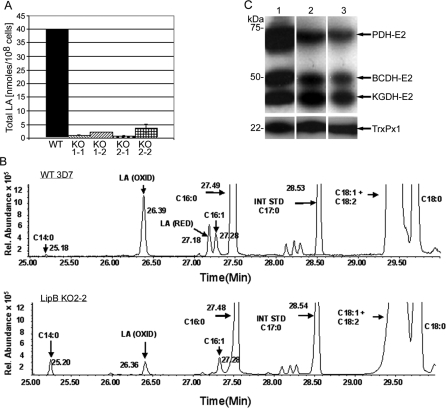
Effect of *LipB* Disruption on Lipoic Acid Levels and PDH-E2 Lipoylation (A) WT 3D7 and *LipB*KO mutants were analysed for their LA content. The data shown were determined in two independent experiments and each sample was measured in triplicate. Wild-type LA levels are around 40 nmoles/10^8^ cells, whereas *LipB*KO lines had drastically reduced LA levels between 0.9 nmoles/10^8^ cells and 2.5 nmoles/10^8^ cells. (B) WT 3D7 and *LipB*KO2–2 mutant were analysed for their fatty acid content. Fatty acids released by acid and base treatment were converted to methyl esters and analysed by GC-MS (see Materials and Methods for details). The figure shows an enlargement of the total ion current (TIC) chromatogram between 25.0 min and 30.0 min in which the fatty acids, including the internal standard (C17:0) at 28.5 min, C14:0 at 25.2 min, C16:1 at 27.3 min, C16:0 at 27.5 min, C18:2, C18:1, and C18:0, as well as the short chain octanoic acid derived LA (oxidised) at 26.4 min, and LA (reduced) at 27.2 min are marked. The levels of myristic acid (C14:0) clearly increase in the *LipB*KO line in comparison to wild-type parasites. (C) Analysis of lipoylation pattern of WT 3D7 and *LipB*KO mutants. Parasite extracts (15 μg) were separated on 4%–12% SDS-PAGE, blotted to nitrocellulose, and subsequently were probed with a rabbit anti-LA antibody as described in Materials and Methods. Lane 1, 3D7 wild-type; lane 2, LipBKO2–1; lane 3, LipBKO2–2. The bands correspond to PDH-E2 (75 kDa), BCDH-E2 (50 kDa), and KGDH-E2 (47 kDa). PDH-E2 lipoylation is greatly reduced in the mutant lines in comparison with wild-type, showing that the decrease in LA levels is primarily due to loss of the cofactor from the apicoplast PDH-E2-subunit. The blot was reprobed with an antibody directed against the 22 kDa 2-Cys peroxiredoxin PfTrx-Px1 as a loading control (Trx-Px1).

**Table 1:**
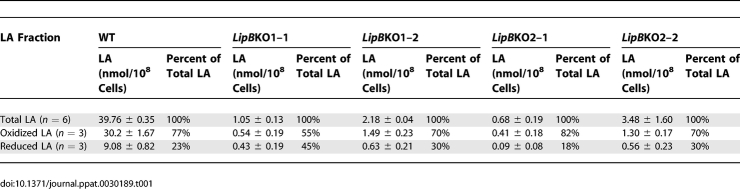
Lipoic Acid Levels in Wild-Type and *LipB*KO P. falciparum

Apart from changes in LA levels, the *LipB* disruption lead to a 5-fold increase in myristate (C14:0) in the *LipB* mutants (from 1.8 nmoles/10^8^ cells to 9 nmoles/10^8^ cells). This is presumably the result of the increased availability of octanoyl-ACP, which is no longer required for LA biosynthesis and so can be further extended to C14 (Figure 5B). Thus, the decreased requirement for LA biosynthesis because of the lack of LipB function generates a surplus of longer chain fatty acids, which can be used for instance for protein modifications such as acylations and lipid biosynthesis. The major overall finding of this part of the study was that the *LipB* disruption leads to a drastic reduction of total LA in the parasites without affecting parasite viability.

From these data it also can be deduced that the level of lipoylation of PDH-E2 should be greatly reduced if not ablated if LipB is the only protein that transfers the LA cofactor to the multienzyme complex in the apicoplast. This was qualitatively analysed by Western blotting using anti-LA antibodies which shows that PDH-E2 lipoylation indeed decreases significantly but does not totally disappear (Figure 5C, compare lane 1 with lanes 2 and 3), suggesting an alternative mechanism that allows for partial PDH-E2 lipoylation in the absence of LipB activity. The E2-subunits of the mitochondrial KADH also show a slight decrease of lipoylation, suggesting that the knockout of the apicoplast *LipB* might also affect the modification of the mitochondrial enzyme complexes.

### Effect on Fatty Acid Biosynthesis and Susceptibility Towards Pro-Oxidants

Apicoplast PDH is thought to be the source of acetyl-CoA for type II fatty acid biosynthesis in apicomplexan parasites [22] and the drastic reduction of LA in the parasites led to the conclusion that the activity of PDH-E2 might be negatively affected and as a consequence the provision of substrates for de novo fatty acid biosynthesis might be reduced. This hypothesis was tested by investigating the sensitivity of the mutants to triclosan, an inhibitor of FabI; one of the enzymes involved in fatty acid elongation in P. falciparum and previously validated as a drug target [23]. However, our data show that the *LipB* disruption does not have any effect on the susceptibility of mutant parasites towards triclosan, possibly suggesting that the residual lipoylation of PDH-E2 is sufficient to provide enough acetyl-CoA to sustain fatty acid biosynthesis at wild type level (Figure 6A).

**Figure 6:**
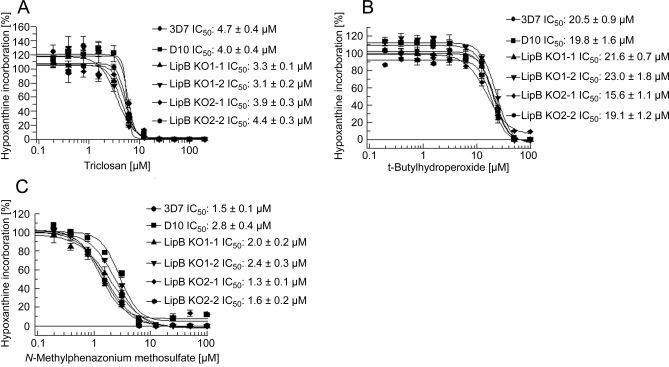
Effect of Triclosan, *tert-*Butylhydroperoxide and *N-*Methylphenazonium Methosulfate on *LipB* Mutants The effect of triclosan (an inhibitor of FabI) (A) and two pro-oxidants (*tert*-butyl hydroperoxide (B) and *N-*methylphenazonium methosulfate (C) on parasite growth were analysed as described in Materials and Methods. The IC_50_ values of wild-type and *LipB*KO mutants do not differ markedly suggesting that LA depletion has no effect on fatty acid biosynthesis and response to pro-oxidants. All three figures show means ± standard errors of three independent experiments.

Another hypothesis requiring investigation was the suggestion that LA might act as a principal antioxidant in the organelles of *Plasmodium*. In order to further substantiate this, the IC_50_ for two pro-oxidants were determined; wild-type and *LipB* null mutants showed no differential susceptibility towards *tert-*butylhydroperoxide and *N-*methylphenazonium methosulfate, respectively (Figure 6B and 6C).

### Can LplA2 Compensate for the Loss of *LipB*?

It was shown previously that *Plasmodium* possess a second functional LplA-like protein, which compensated growth of a bacterial strain lacking both *LipB* and *LplA,* but which was unable to compensate growth of a *LipB* deficient bacterial line in a previous study [14]. It was suggested that LplA2 is not able to replace LipB function in the bacteria because of their distinct substrate specificities. In this study we expressed three different expression constructs in *LipB* and *LipB/LplA* deficient Escherichia coli lines, respectively [24,25]. The constructs used here were full length at their C-termini and included a C-terminal tag as opposed to the construct expressed in the previous study. The N-terminus of the three constructs differed because the deduced amino acid sequence has an N-terminal extension of 28 amino acids when compared to E. coli LplA which potentially encodes an N-terminal targeting sequence. It is possible that such N-terminal targeting sequences interfere with efficient expression or function of the heterologous parasite protein in the prokaryotic expression system and therefore it was decided to analyse three different expression constructs of LplA2 (as outlined in the Materials and Methods section) in the complementation assay. In our hands all three constructs complemented the growth of the *LipB* deficient bacteria (Figure 7A), suggesting that the protein can replace LipB function. These data are in contrast to those obtained by [14] but might be explained by differences in expression plasmids and constructs used in the two different studies. This does not exclude the possibility that LplA1 and LplA2 proteins have differential, albeit somewhat overlapping, substrate specificities or activity profiles through the developmental cycle of P. falciparum. The LplA2 constructs also compensated for the growth defect of TM136, a bacterial line deficient in *LplA* and *LipB* (Figure 7B) [25].

**Figure 7:**
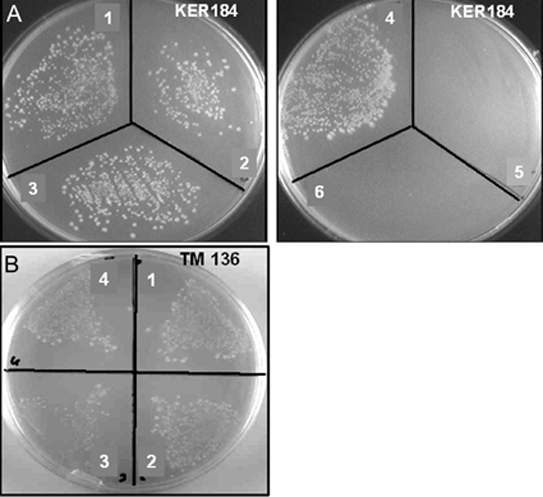
LplA2 Functional Complementation of *LipB* and *LplA/LipB* Deficient E. coli The functionality of LplA2 was analysed by complementing two bacterial lines with three different expression constructs as described in Materials and Methods. (A) Growth of KER 184 in the presence and absence of LplA2 expression constructs as described in Materials and Methods. Key: 1, pASK-IBA3-LplA2_fl_; 2, pASK-IBA3-LplA2-S1; 3, pASK-IBA3-LplA2-S2; 4, pASK-IBA3-LplA1; 5, pASK-IBA3; 6, untransformed (B) Growth of TM 136 in the presence of LplA1 and LplA2 expression constructs. Key: 1, pASK-IBA3-LplA2_fl_; 2, pASK-IBA3-LplA2-S1; 3, pASK-IBA3-LplA2-S2; 4, pASK-IBA3-LplA1.

### Localisation of LplA2

A clear prediction of LplA2′s localisation is not possible and therefore this was analysed by expressing a C-terminally green fluorescent protein (GFP)-tagged full-length LplA2 protein in the erythrocytic stages of P. falciparum. The results suggest that LplA2-GFP is targeted to two distinct organelles—one of which is clearly the mitochondrion as LplA2-GFP colocalises with Mitotracker (Figure 8). Given the close association of the second organelle to the mitochondrion we suggest that this is likely to be the apicoplast. This was corroborated by immunofluorescence studies using antibodies raised against LplA2 and apicoplast lipoamide dehydrogenase (aLipDH; aE3; P. J. McMillan and S. Müller, unpublished data). The results show that in some parasites both proteins clearly colocalise, supporting its localisation in the apicoplast (Figure 9). However, the localisation of LplA2 is not that straightforward. In some parasites we clearly observe colocalisation with either Mitotracker or, in the immunofluorescence study, with aLipDH, whereas in others the staining is present in both organelles. Analysing the distribution in 46 distinct parasites from the immunofluorescent experiment resulted in the following distribution: 19.6% of LplA2 colocalised with aLipDH and thus is apicoplast located; 53% were found in an organelle distinct to the apicoplast but likely to be the mitochondrion, and 28 % showed staining of both organelles. Similarly, the LplA2-GFP expressing parasites were analysed (50 parasites) and 15% of staining was likely to be apicoplast (distinct from the mitochondrion stained with Mitotracker), 68% were found to have LplA2-GFP in the mitochondrion and 18% showed staining in both organelles. These data indicate that LplA2 is dually targeted within the parasites albeit the precise mechanisms governing this distribution need to be analysed in future studies.

**Figure 8:**
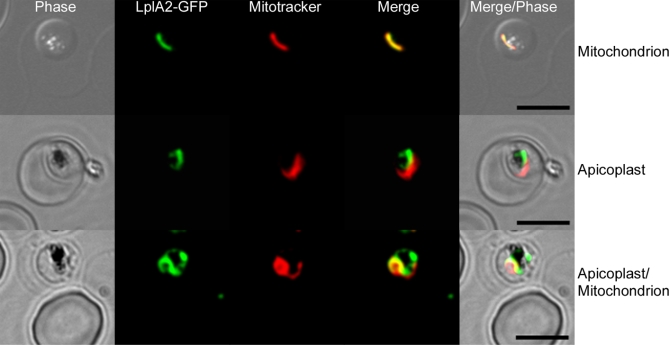
Localisation of LplA2-GFP The localisation of LplA2 cannot be predicted and therefore it was analysed by localising a C-terminally GFP-tagged protein in P. falciparum erythrocytic stages. Expression of the GFP-tagged protein in P. falciparum 3D7 was analysed by fluorescence light microscopy. The parasites were costained with Mitotracker^TM^ Ros (Molecular Probes) to assess whether LplA2-GFP colocalises with the parasite's mitochondrion. In panel 1 LplA2 is clearly colocalising with the mitochondrion. However, as is obvious from panel 2 LplA2-GFP localised also to an organelle close to the mitochondrion but clearly distinct from it. In panel 3 LplA2-GFP colocalises with the mitochondrion but also is present in a second organelle closely associated with the mitochondrion. Scale bars: 5 μm.

**Figure 9:**
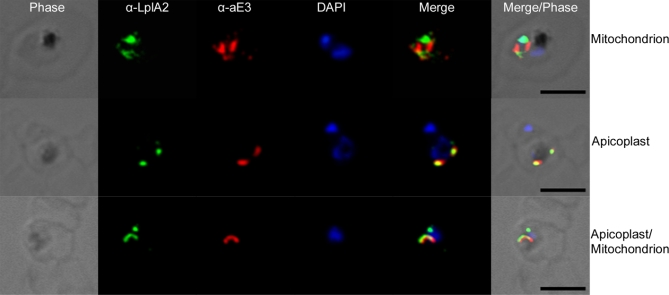
Localisation of LplA2 by Immunofluorescence Studies Immunofluorescent analyses were performed on P. falciparum 3D7 using anti-LplA2 and anti-aLipDH (aE3) antibodies (specifically staining the apicoplast of the parasites) as outlined in the Materials and Methods section. The data suggest a similar distribution of LplA2 as that shown by expressing a C-terminally tagged LplA2-GFP construct in the parasites. Panel 1 shows staining of an organelle distinct from the apicoplast, probably being the mitochondrion. Panel 2 shows costaining of both antibodies, suggesting LplA2 is located in the apicoplast, and panel 3 shows staining of both the apicoplast and potentially the mitochondrion. Scale bars: 5 μm.

Overall, the functionality and localisation studies of LplA2 strongly support our hypothesis that LplA2 indeed compensates for the loss of LipB function, although it appears that its ability to utilise the octanoyl-ACP substrate provided by fatty acid biosynthesis might not be as efficient as it is by LipB given the extensive loss of protein-bound LA and the reduction of lipoylation of PDH-E2.

## Discussion

Lipoic acid metabolism in apicomplexan parasites is distributed between mitochondrion and apicoplast [11,12]. Both organelles lipoylate their KADHs independently, with de novo biosynthesis confined to the apicoplast and salvage solely found in the mitochondrion of the parasite [13–15].

Given this background it was surprising that the *LipB* gene, which encodes the principal LA protein ligase in the apicoplast, can be disrupted without negatively affecting *P. falciparum* growth. This was entirely unexpected because it was thought that LipB activity is absolutely required for the parasites to lipoylate PDH-E2, which in turn is necessary to provide acetyl-CoA for fatty acid biosynthesis operating in the organelle [22]. In fact, down regulation of ACP expression using a Tet-inducible conditional knockout system in T. gondii revealed that one major function of apicoplast located type II fatty acid biosynthesis is to provide octanoyl-ACP for the lipoylation of PDH-E2 in these related parasites [15]. These data indirectly suggest that salvage of LA and its subsequent ligation to the PDH-E2 is not an alternative for the apicoplast located LA de novo biosynthesis pathway in *Toxoplasma*. This is in agreement with data on bacterial LipB, which does not accept free LA as a substrate [6,26]. Thus, even if exogenous LA would be taken up into the apicoplast of *Toxoplasma* it would require the presence of a LplA-like protein to guarantee ligation of the cofactor to the apo-PDH-E2. Analyses of a gene identified in the ToxoDB database potentially encoding LplA2 (gene locus: 83.m01296) showed that the identity between the deduced amino acid sequences of the *Toxoplasma* and *Plasmodium* deduced amino acid sequences was modest, with less than 10% identity. This is in contrast to potential LplA2 encoding genes in other *Plasmodium* species but also other apicomplexans, such as *Theileria*, which contain highly conserved orthologues of *P. falciparum* LplA2 (similarities between 58% and 37%). In addition, highly conserved amino acid motifs necessary for substrate interaction and activation appear to be absent from the potential T. gondii LplA2 [27–29]. Therefore, it appears that the lipoylation pathways in *Plasmodium* and *Toxoplasma* differ to some extent.

The LA content of the *LipB* mutant parasites was analysed by GC-MS which revealed that the *LipB* mutants had a drastic reduction (∼90%) of LA compared to wild-type parasites. Concomitantly, the levels of myristate (C14:0) increased about 5-fold in the mutant parasites compared to wild-type parasites, which could potentially have implications such as the levels of protein lipidations or specific lipid species in the mutant parasite lines [30]. Despite the severe loss of LA, the *LipB* mutant parasites showed a faster growth phenotype which was primarily attributable to accelerated progression through the intraerythrocytic cell cycle. It is conceivable that the increased availability of endogenously generated myristate changes the acylation state of regulatory proteins and affects their activity and/or distribution, which potentially contributes to the observed phenotype. However, these speculations have to be further substantiated in future analyses of the *LipB* mutants. Another aspect is that the loss of LA has an impact on the parasites' capacity to defend themselves against oxidative stress considering that one of the most discussed roles of LA is its redox activity and its potential as antioxidant [31,32]. However, the *LipB* mutants appear to be unaffected in their susceptibility to exogenous and endogenous oxidants despite the significant loss of LA. This potentially could be explained by a compensatory upregulation of alternative antioxidants in these mutant parasites—a hypothesis that also needs further investigation.

The observed reduction of LA in the parasites also implies that PDH-E2 lipoylation should be greatly reduced; by using an anti-LA antibody this was confirmed by Western blotting. The reduced lipoylation appears to not negatively affect de novo fatty acid biosynthesis as shown by the fatty acid analyses data (reduction of LA biosynthesis leads to increased levels of longer chain fatty acids). In addition, mutant and parent parasite lines showed similar susceptibilities to the supposed FabI inhibitor triclosan [21]. This shows that the reduction of PDH-E2 lipoylation does not affect PDH activity as severely as originally believed and that the multienzyme complex still provides sufficient acetyl-CoA to sustain fatty acid biosynthesis. Previous studies on E. coli PDH showed that the loss of one or two of the three lipoyl-domains of the bacterial PDH-E2 subunit does not cause significant changes in PDH activity demonstrating that under-lipoylation does not necessarily yield a catalytically incompetent PDH complex [33].

The fact that PDH-E2 is at all lipoylated is surprising given that LipB was thought to be the principal LA protein ligase present in the apicoplast. However, we have shown in this study that an alternative pathway that allows lipoylation of PDH-E2 is provided by LplA2, a second LA protein ligase like protein, identified in the genome of several *Plasmodium* species [14] and a number of other apicomplexan parasites. However, *Toxoplasma* appears to lack a LplA2 orthologue—a gene potentially encoding LplA2 lacks amino acid motifs essential for a functional LplA protein. The functionality of *Plasmodium* LplA2 was corroborated in this study and it was shown that different expression constructs complement the growth defect of bacteria deficient in *LipB* and *LipB/LplA*, supporting that the protein compensates for both LplA and LipB in this bacterial expression system. This is in contrast to the findings of [14] who suggested that LplA2 can replace mitochondrial LplA1 only, and not LipB. However, the expression constructs used in the previous study differ considerably from the ones used in this study, which might explain these different results. Furthermore, it needs to be emphasised that LplA2 seems to be less efficient in using octanoyl-ACP as a substrate to lipoylate the PDH-E2 subunit as shown by the large loss of total LA and the under-lipoylation of PDH-E2. This clearly suggests that the substrate specificities of LipB and LplA2 might differ and studies to fully characterise LplA2 biochemically and its precise role for parasite survival are currently underway. The difference in substrate specificity between LipB and LplAs is, however, not very surprising and has been previously shown for the bacterial enzymes [6]. In fact, overexpression of LipB in E. coli render them insensitive towards selenolipoic acid, suggesting that LipB cannot use exogenously supplied LA or its derivatives as substrates [6,26].

The localisation of LplA2 cannot be reliably predicted and therefore the full-length protein C-terminally tagged with GFP was expressed in the erythrocytic stages of P. falciparum. The results that we obtained were intriguing because the GFP fluorescence was observed in both the mitochondrion and a closely associated organelle likely to be the apicoplast. In order to exclude that these results were attributable to the over expression of the LplA2-GFP-fusion protein we also performed immunofluorescence studies on wild-type parasites. The anti-LplA2 antibody used detected a protein that either colocalised with an apicoplast marker (aLipDH), was closely associated with the apicoplast marker, or was observed in both organelles. This implies that LplA2 is dually targeted to mitochondrion and apicoplast supporting the hypothesis that the loss of LipB functionality can be compensated by LplA2. Dual targeting has been shown previously in P. falciparum for the metalloprotease falcilysin [34] and potential mechanisms by which this is achieved or governed have been discussed by Ralph [35]. In organisms that contain plastid and mitochondrion dual targeting is not unusual, particularly for those proteins involved in biological processes that are found in both organelles [36,37]. Most proteins targeted to the two organelles are nuclear-encoded and they possess certain targeting signals that are not always predictable using bioinformatics approaches [38]. It has been suggested that not only the primary amino acid sequence of a protein is involved in the control of dual targeting but that also untranslated regions of the mRNA play a role in the process [37,39]. In apicomplexan parasites the posttranslational trafficking of apicoplast and mitochondrial proteins differs considerably from those in plants—mitochondrial proteins are delivered via the cytosol to their destination whereas the apicoplast targeting is through the secretory pathway [40]. Therefore it has to be assumed that specific mechanisms allow for dual targeting of apicomplexan proteins to both organelles. Recently, a study by Pino and colleagues showed that the nature of the signal peptide affects targeting of a number of unrelated proteins to both mitochondrion and apicoplast in T. gondii [41]. It is well possible that similar mechanisms occur in *Plasmodium* and the fact that lipoylation is an essential process in both organelles might be the reason for the dual targeting of LplA2 that we observed in this study.

Overall, this study shows that redundancies exist between LA protein ligases in the malaria parasite P. falciparum, which appear to be achievable through dual targeting of LplA2. The accelerated progression through the cell cycle during intraerythrocytic growth of *LipB* mutants implies that the lack of LA and LipB is affecting cell cycle control mechanisms. Future studies will elucidate the underlying reasons for the rapid progression through the intraerythrocytic cycle and analyse whether there are growth and developmental impairments during other life cycle stages of the *LipB* mutant parasites.

## Materials and Methods

### Materials.

Albumax II and RPMI 1640 were obtained from Invitrogen Corporation, UK. Irgasan (triclosan), *tert*-butylhydroperoxide and *N-*methylphenazonium methosulfate were purchased from Sigma-Aldrich, UK. The Immobilon^TM^ Western Chemiluminescent HRP Substrate was obtained from Millipore, UK. Anti-LA rabbit polyclonal antibody was supplied by Calbiochem and the anti-rabbit IgG (H+L), HRP conjugate was from Promega. [α-^32^P]-ATP (Adenosine 5′-triphosphate [α-^32^P], EasyTides, specific activity: 3,000 Ci/mmol) was purchased from Perkin-Elmer. [8-^3^H]-hypoxanthine (specific activity: 10–30 Ci/mmol) was from GE Healthcare, UK. All restriction enzymes were obtained from New England Biolabs. WR99210 was generously provided by Dr Jacobus, Jacobus Pharmaceuticals, USA. The vector pASK-IBA3 was purchased from Institut für Bioanalytik, Germany. The *LipB*-deficient (KER 184) and *LipB/LplA* deficient (TM136) bacterial strains were a kind gift from Dr John Cronan (University of Illinois at Urbana-Champaign, USA). Plasmids pHH1and pCDH3/4, PfHSP86 5′-pDONR4/1, and PfCRT 5′-pDONR4/1 were kind gifts from Professor A. F. Cowman (The Walter and Eliza Hall Institute for Medical Research, Melbourne, Australia) and Professor G. I. McFadden (University of Melbourne, Australia), respectively.

### Knockout and expression constructs of *P. falciparum LipB*.

The expression and knockout fragments of the *P. falciparum LipB* gene were amplified from P. falciparum 3D7 and D10 genomic DNA using *Pfx* Supermix (Invitrogen). The specific oligonucleotide primers 5′-GCGC**AGATCT**AATAAAATAAACCTGCTTGTAC-3′ (sense) and 5′-GCGC**CTCGAG**(TTA)TTTATCCTTATAAAAGATACC-3′ (antisense) with the BglII and XhoI restriction sites, respectively, in bold, and an artificial stop codon (in brackets) within the antisense oligonucleotide, were used to generate the 999 bp insert equivalent to nucleotides 4–1,002 of the *PfLipB* open reading frame for the *PfLipB*KO1 construct. The PCR product was subcloned into the TOPO-Blunt PCR cloning vector (Invitrogen) and its sequence was verified (The Sequencing Service, University of Dundee, UK, http://www.dnaseq.co.uk/) before it was cloned into the P. falciparum transfection plasmid pHH1 [19]. The second construct comprises nucleotides 304–1,002 of the open reading frame missing the potential bipartite targeting sequence. The insert for *PfLipB*KO2 was amplified using the sense primer 5′-GCGC**AGATCT**ATTATGAAAAATAAAAATGAAGTACAAATATCAAATCATTTAG-3′ and the same antisense primer as for *PfLipB*KO1. The 699 bp product was subcloned into TOPO-Blunt as described above for sequence verification before being cloned into pHH1.

### Parasite culture, transfection, determination of IC_50_, and parasite growth rate.

P. falciparum 3D7 (The Netherlands) and P. falciparum D10 (Papua New Guinea) were cultured according to Trager and Jensen [42] with modifications in human erythrocytes, RPMI 1640 containing 11 mM glucose, with the addition of 0.5% Albumax II. The parasites were maintained under an atmosphere of reduced oxygen (1% oxygen, 3% CO_2_, and 96% nitrogen) at 37 °C. Parasites were synchronised using sorbitol according to Lambros and Vanderberg [43].

Transfection of *PfLipB*KO1-pHH1, *PfLipB*KO2-pHH1, *HSP86-LplA2-GFP*-pCHDR, and *CRT-LplA2-GFP*-pCHDR into P. falciparum erythrocytic stages was performed as described previously [44,45]. WR99210 resistant parasites appeared between 40 and 60 days after transfection. Parasites were cloned by limiting dilution according to Kirkman et al. [46].

The effect of triclosan, *N-*methylphenazonium methosulfate, and *tert*-butylhydroperoxide on P. falciparum erythrocytic stages was determined by measuring the incorporation of [^3^H]-hypoxanthine in the presence of increasing drug concentrations (0.5 μM to 100 μM) according to [47].

Relative parasite growth rates were determined using the method of Sanders et al. [21]. Parasite cultures containing mainly ring stages were synchronised twice within 4 h using sorbitol [43]. Parasite density was determined and the culture was diluted to 0.5% parasitaemia, 5% haematocrit. Cultures were maintained under an atmosphere of reduced oxygen at 37 °C and medium was refreshed every 24 h. Cultures were diluted 5-fold at 48 h intervals and growth was monitored by Giemsa-stained thin blood smears every 24 h. For each determination of percentage parasitaemia the number of infected erythrocytes per 1,000 erythrocytes was recorded.

### Concentration of trophozoite-infected erythrocytes using MACS columns.

Cultured parasites were enriched for late-stage forms by using the VarioMACS separator and CS MACS columns (Miltenyi Biotec). The columns were equilibrated with MACS buffer (PBS supplemented with 0.5% (w/v) BSA, 2mM EDTA) for 5 min and rinsed with 60 ml of MACS buffer. The equivalent of 100 ml cultured P. falciparum was resuspended in MACS buffer and applied to the column. Following flow-through of the cell-suspension, the column was washed with 50 ml MACS buffer. The column was then removed from the magnetic field of the VarioMACS separator and the late-stage parasitised erythrocytes were eluted from the column using 30 ml MACS buffer. The suspension was centrifuged and the cells were resuspended in complete medium and returned to culture conditions for 30 min prior to harvesting by saponin lysis. The typical yield from this procedure was 1–2 × 10^8^ isolated late-stage parasites.

### Preparation of genomic DNA and protein from P. falciparum.

The parasites were liberated from erythrocytes by saponin lysis [48] and genomic DNA was isolated using the QIAamp DNA Mini Kit (Qiagen). Protein extracts were prepared from saponin-isolated parasites by resuspending the pellets in lysis buffer (100 mM HEPES (pH 7.4), 5 mM MgCl_2_, 10 mM EDTA, 0.5% (v/v) TritonX-100, 5 μg/ml RNAse, 1 mM phenylmethylsulphonyl fluoride, 1 mM benzamidine, 2 μg/ml leupeptin, 10 μM E-64, 2 μM 1,10-phenanthroline, 4 μM pepstatin A) followed by three cycles of freeze/thawing and sonication in a sonicating water-bath (Fisherbrand). Protein concentrations were determined using the Bradford assay [49].

### Southern blot analyses.

One μg of genomic DNA was digested with NdeI, separated on a 0.8% agarose gel, and blotted onto positively charged nylon membrane (GE Healthcare) using standard methods [50]. The blot was probed with the *LipB* coding sequence. Radioactive probes were made using the MegaPrime Labelling Kit from GE Biosciences and [α-^32^P]-ATP (Adenosine 5′-triphosphate [α-^32^P], EasyTides, Specific Activity: 3,000 Ci/mMole) from Perkin-Elmer following the manufacturer's recommendations. The membrane was prehybridised (0.5% (w/v) SDS, 5 × Denhardt's solution, 100 μg/ml salmon sperm DNA, 0.1% (w/v) sodium pyrophosphate) for 2 h at 60 °C before addition of the probe. Hybridisation was then allowed to proceed at 60 °C over night. Membranes were washed once in 6× SSC, 0.1% (w/v) SDS at 60 °C for 20 min, and then twice in 2× SSC, 0.1% (w/v) SDS at 60 °C for 10 min. Membranes were exposed to Kodak film for several days before development depending on the activity of the probe used.

### Western blot analyses.

To determine lipoylation of KADH-E2 subunits in P. falciparum, protein extracts of parasites were subjected to Western blotting. Briefly, 15 μg of each sample was separated on a 4%-12% SDS-PAGE (Invitrogen) and then blotted onto nitrocellulose (Schleicher and Schüll), using standard techniques [50]. The blot was incubated with a rabbit anti-LA antibody (Calbiochem) at a dilution of 1:500 and the secondary anti-rabbit IgG (H+L), HRP conjugate (Promega) at a dilution of 1:10,000 before being developed using the Immobilon^TM^ Western Chemiluminescent HRP Substrate (Millipore). Similarly, blots of wild-type parasite extracts were also probed with antibodies against BCDH-E2 (raised in rabbit), KGDH-E2 (raised in rat) and H-protein (raised in rabbit) of P. falciparum (all generated by Eurogentec, Belgium) at dilutions of 1:5,000, 1:100 and 1:2,000, respectively.

### Quantitation of lipoic acid by gas-chromatography mass-spectrometry.

Samples for LA analyses were prepared from late trophozoites prepared using the MACS columns described above. The LA detection method was modified from that of Pratt and colleagues [51]. LA determinations were done in triplicate, along with a parallel control of standards, all containing an internal standard of heptadecanoic acid (10 nmol). Experimental details for the determination of LA will be published elsewhere (T. K. Smith, in preparation). Briefly, total LA was determined by acid hydrolysis of freeze-dried parasite pellets to release protein bound fatty acids. Reduction with NaBH_4_ and methylation with methyl iodide of the sulphydryl groups under basic conditions was followed by organic extraction of all fatty acids, including the methylated LA. The dried fatty acids were converted to fatty acid methyl esters (FAME) with diazomethane and stored dried at −20 °C until subjected to GC-MS. Quantification of oxidised LA was as above except no reduction or methylation of the sulphydryl groups is required. Free LA was extracted from a freeze-dried cell pellet with organic solvents which were checked for protein by analysis of SDS-PAGE and staining, prior to treatment with methanolic HCl and conversion to fatty acid methyl esters as above.

Analysis of the FAMEs was conducted on a Hewlett Packward 6890–5973 system equipped with a ZB-5 30M × 0.25 mm (I.D.) column. The electron impact ionization/quadrupole mass detector was programmed to monitor selected ions for all FAMEs m/z 123, heptadecanoic acid m/z 284, oxidised LA m/z 220, reduced and methylated m/z 250, with typical elution times of oxidised LA, 26.4 min; reduced and methylated LA, 27.2 min; and heptadeconic acid (C17:0), 28.5 min. Molar response factor for oxidised LA and methylated reduced LA versus heptadecanoic acid were determined to estimate the LA content. In addition, the total ion current (TIC) chromatograms were analysed for changes in other longer chain fatty acids.

### Complementation of *LipB* and *LipB/LplA*-deficient E. coli by LplA2.

The functionality of three expression constructs of LplA2, generated in pASK-IBA3, was analysed. The following anti-sense primer 5′-GCGCGC**GGTCTCAGCGCT**TAGAAAATATGTTGGTATATCGTAATACC-3′ was used to amplify all three constructs. In combination with the sense primer 5′-GCGCGC**GGTCTCGAATG**AGAATTATAAAGTGCCTGGATC-3′ a 1,152 bp fragment was amplified corresponding to the full length gene. The sense primer 5′-GCGCGC**GGTCTCGAATG**AAAAAAATAAACATTCTTTATTTTATTGATGTCAGC-3′ generated a truncated fragment from nucleotide 79–1,152 (S1 construct). The third fragment (S2) amplified using 5′-GCGCGC**GGTCTCGAATG**AATGAGTCCAAAGGAAACGAATGC-3′ corresponds to nucleotide 235–1,152 bp. All primers contained a BsaI restriction site (boldface) to allow directional cloning into pASK-IBA3. The constructs were amplified from P. falciparum 3D7 genomic DNA using *Pfx* Supermix (Invitrogen) and were initially cloned into TOPO-Blunt PCR cloning vector (Invitrogen) for sequence verification. Subsequently, they were subcloned into pASK-IBA3 and transformed into KER 184 and TM 136 [24,25] to assess whether they complement the growth defect of the bacterial lines when grown on minimal medium agar plates.

### Localisation of LplA2.

To analyse the localisation of LplA2, the full length *LplA2* gene was amplified from P. falciparum 3D7 genomic DNA using the sense primer 5′-CACCATGAGAATTATAAAGTGCCTGG-3′ and antisense primer 5′-TAGAAAATATGTTGGTATATCGTAATACC-3′. The PCR product was cloned directionally into the plasmid pENTR/D-TOPO (Invitrogen) and the sequence was verified by sequencing. The cloning of the constructs was performed as described by van Dooren et al. [52]. The destination plasmid used was pCHDR-3/4 which contains the human dihydrofolate reductase (*hDHFR*) as a selectable marker. Two entry plasmids were used that differed in their promoter regions. The *PfHsp86* 5′-pDONR4/1 contains the P. falciparum heat shock protein 86 5′ UTR and the *PfCRT* 5′-pDONR4/1 possesses the P. falciparum chloroquine resistance transporter 5′UTR. The C-terminal GFP-tag was provided by *GFP*-pENTR2/3. Thus, four plasmids (either *PfHspP86*-pENTR4/1 or *PfCRT*-pENTR4/1, *LplA2*-pENTR/D-TOPO, *GFP*-pENTR2/3, and pCHDR-3/4) were incubated in the LR MultiSite cloning reaction according to manufactures guidelines (Invitrogen) which resulted in the generation of two *LplA2* constructs (*Hsp86-LplA2-GFP*-pCHDR and *CRT-LplA2-GFP*-pCHDR). Constructs were transfected as described above and parasites resistant to WR99210 were analysed using an Axioskop-2 mot plus microscope (Zeiss) equipped with a Hamamatsu C4742–95 CCD camera.

Fixations of wild-type 3D7 parasites for subsequent immunofluorescence analyses were carried out according to [53]. The primary antibodies raised against LplA2 (in a rat, Eurogentec) and against apicoplast lipoamide dehydrogenase (aLipDH; aE3) (in a rabbit, Eurogentec) were diluted in 3% (w/v) BSA in PBS at 1:500 and 1:200, respectively. Secondary antibodies (anti-rat conjugated with Alexa fluor 488 and anti-rabbit conjugated with Alexa fluor 594, Molecular Probes) were applied at 1:500 dilution in 3% (w/v) BSA in PBS for 1 h at 4 °C. DAPI at 0.5 μg/ml (Sigma) was added to the secondary antibody for 1 min and was then washed off as before. The slides were mounted with 2.5% (v/v) DAPCO in 50% (v/v) glycerol (Sigma) and were analysed using an Axioskop-2 mot plus microscope (Zeiss) equipped with a Hamamatsu C4742–95 CCD camera.

## Supporting Information

### Accession Numbers

The accession numbers and ID numbers of the genes (obtained from the NCBI-protein database) described in this study are as follows: Bos taurus lipoate-activating enzyme (BAB40420), Bos taurus lipoyltransferase (BAA24354), E. coli (K12) LplA (NP_418803), E. coli (K12) LipB (NP_415163), *P. berghei* LplA2 (XP_679932; CAH95194), P. chabaudi LplA2 (XP_745010; CAH79244), P. falciparum ACP (XP_001349595; AAC71866), P. falciparum apicoplast lipoamide dehydrogenase (XP_001349365; CAD51214), P. falciparum BCDH-E2 (XP_001351112; CAB38991), *P. falciparum* enoyl-ACP reductase (FabI) (XP_966137; CAG25389), P. falciparum H-protein (XP_001348010; AAN35923), P. falciparum KGDH-E2 (XP_001349947; CAD52355), P. falciparum LipA (XP_001350160; CAD52569), P. falciparum LipB (XP_001349288; CAD51137), P. falciparum LplA1 (XP_001349882; CAD52290), P. falciparum LplA2 (XP_001352107; CAD51918), P. falciparum PDH-E2 (XP_001347486; AAN35399), P. knowlesi LplA2 (gene ID PlasmoDB: PKH_072080), P. vivax LplA2 (PlasmoDB gene ID: Pv099590), P. yoelii LplA2 (XP_730272 ; EAA21837), *Theileria parva* strain Ankara LplA2 (XP_954802), T. gondii ACP (AAC63956; AAC63953), T. gondii LplA2 (ToxoDB gene ID 83.m01296).

## Abbreviations

ACP: acyl carrier protein
anti-LA: antibody directed against protein-bound lipoic acid
BCDH: branched chain α-keto acid dehydrogenase
BCDH-E2: BCDH transacylase subunit
FAME: fatty acid methyl ester
GC-MS: gas-chromatography mass-spectrometry
GCS: glycine cleavage system
GFP: green fluorescent protein
KADH: α-keto acid dehydrogenase complex
KGDH: α-keto glutarate dehydrogenase
KGDH-E2: KGDH succinyltransferase subunit
LA: lipoic acid (6,8-thioctic acid)
LplA1: lipoic acid protein ligase A1
LplA2: lipoic acid protein ligase A2
LipB: octanoyl-[acyl carrier protein]: protein N-octanoyltransferase
PDH: pyruvate dehydrogenase
PDH-E2: PDH acetyltransferase subunit
